# Maritime Small Target Image Detection Algorithm Based on Improved YOLOv11n

**DOI:** 10.3390/s26010163

**Published:** 2025-12-26

**Authors:** Zhaohua Liu, Yanli Sun, Pengfei He, Ningbo Liu, Zhongxun Wang

**Affiliations:** 1 School of Physics and Electronic, Yantai University, Yantai 264005, China; 2150034422@s.ytu.edu.cn (Z.L.);; 2 Shandong Provincial Data Open Innovation Application Laboratory for Advanced Smart Grid Technologies, Yantai University, Yantai 264005, China; 3 Aviation Basic College, Naval Aviation University, Yantai 264001, China; 4 Information Fusion Institute, Naval Aviation University, Yantai 264001, China

**Keywords:** maritime small target detection, YOLOv11n, deep learning, infrared and visible light images

## Abstract

Aiming at the problems of small-sized ships (such as small patrol boats) in complex open-sea backgrounds, including small sizes, insufficient feature information, and high missed detection rates, this paper proposes a maritime small target image detection algorithm based on the improved YOLOv11n. Firstly, the BIE module is introduced into the neck feature fusion stage of YOLOv11n. Utilizing its dual-branch information interaction design, independent branches for key features of maritime small targets in infrared and visible light images are constructed, enabling the progressive fusion of infrared and visible light target features. Secondly, RepViTBlock is incorporated into the backbone network and combined with the C3k2 module of YOLOv11n to form C3k2-RepViTBlock. Through the lightweight attention mechanism and multi-branch convolution structure, this addresses the insufficient capture of tiny target features by the C3k2 module and enhances the model’s ability to extract local features of maritime small targets. Finally, the ConvAttn module is embedded at the end of the backbone network. With its dynamic small-kernel convolution, it adaptively extracts the contour features of small targets, maintaining the overall model’s light weight while reducing the missed detection rate for maritime small targets. Experiments on a collected infrared and visible light ship image dataset (IVships) and a public dataset (SeaShips) show that, on the basis of increasing only a small number of parameters, the improved algorithm increases the mAP@0.5 by 1.9% and 1.7%, respectively, and the average precision by 2.2% and 2.4%, respectively, compared with the original model, which significantly improves the model’s small target detection capabilities.

## 1. Introduction

Maritime target detection serves as the core technical support for maritime surveillance, maritime rights protection, and intelligent shipping systems. Its detection accuracy and real-time performance directly determine the response efficiency of maritime tasks. However, in scenarios such as maritime target detection using infrared and visible light images, ship targets often appear as small targets with low pixel proportions and sparse feature information. Traditional algorithms tend to suffer from false detection and missed detection due to issues like insufficient receptive fields and the easy suppression of small target features, making it difficult to meet the needs of practical applications.

Preliminary explorations have been conducted in the field of deep learning-based maritime target detection for small ship image detection. Early studies mostly relied on two-stage detection frameworks to improve the detection accuracy for small ships. For instance, Tan et al. [1] modified Faster R-CNN by incorporating soft NMS and focal loss; Jian et al. [2] improved the two-stage target detection model of Mask R-CNN for marine ship detection. However, constrained by the large computational costs of two-stage frameworks and the lack of targeted optimization for fusion strategies, it remains challenging to balance accuracy and efficiency in small target detection scenarios.

With the gradual rise of one-stage detection algorithms, improved methods based on the YOLO series [3,4,5,6,7] have become a research focus in the field of small ship detection. For example, Xie et al. [8] focused on the SAR ship detection task, fused the coordinate attention mechanism into the YOLOv5 deep learning detector, enhanced the feature extraction and localization capabilities for ship targets, and meanwhile balanced the model’s real-time performance and detection accuracy. Liu et al. [9] proposed the CGSE-YOLOv5s algorithm, which improves the model’s capabilities in infrared ship detection in complex nearshore scenes by integrating contrast-limited adaptive histogram equalization with Gaussian filtering, replacing the feature pyramid network module, and introducing an efficient channel attention mechanism. Zheng [10] et al. optimized the structure of YOLOv7x and added a CBAM module to enable the model to focus more on small targets in images. Wang et al. [11] put forward an improved YOLOX_s ship target detection algorithm, introducing the focal loss function to effectively reduce the false detection rate. Li et al. [12] proposed the YOLO-Vessel ship detection model, which innovatively incorporates the ELAN-OD Conv backbone network structure based on YOLOv7 to enhance the feature extraction capabilities in complex backgrounds; they simultaneously introduced the idle depth structure and ASFF Predict network structure into the head network to improve the detection performance for small and medium-sized ship targets. Gong et al. [13] proposed Ship-YOLOv8, which improved the model’s ability to recognize long-distance ships (similar to small targets). Zhang et al. [14] proposed the Ship-FireNet algorithm based on YOLOv8n, which achieves lightweight and accurate ship fire detection by introducing GhostnetV2-C2F, spatial-channel construction convolution (SCConv), and omni-dimensional dynamic convolution and constructing a ship fire dataset. Zhao et al. [15] proposed MFAFNet, a multi-functional attention fusion network designed specifically for infrared remote sensing scenarios, which includes a functional interaction fusion module, a patch attention module and an asymmetric context fusion module, and it improved the detection performance on infrared small targets by integrating features and capturing different receptive field scales.

Subsequently, explorations on the improvement of lightweight models have gradually unfolded, and researchers have put forward higher requirements for the inference speeds of models. For example, Zhao et al. [16] proposed a lightweight SAR small ship detection network, LWSARDet, based on the YOLOv5 framework. By constructing a feature extraction module, designing a lightweight detection head, and proposing a corresponding loss function, it addresses challenges such as high-frequency noise interference and computing power limitations in SAR ship detection under complex sea conditions. Zhao et al. [17] proposed a maritime target recognition and localization system based on a lightweight neural network, which reduces the missed detection rate of the YOLO model in detecting small targets. Bao et al. [18] developed a lightweight enhanced detection model named YOLO-LDFI, which incorporated four architectures, including linear deformable convolution (LDConv), to improve the model’s positioning ability for small ship targets. Sun et al. [19] proposed an infrared ship detection network (IRSD-Net) based on the YOLOv11n framework, which significantly enhanced the model’s ability to detect small-scale ships in cluttered backgrounds and difficult scenarios. These works have verified the application potential of lightweight models in small ship detection scenarios. The work of existing scholars has, to a certain extent, advanced the research progress of maritime small target image detection, but there are still the following shortcomings:

1. Although some improved methods perform well on specific datasets, their generalization ability under different modal data, cloud-shaded, or nighttime scenarios still needs further verification.
2. The balance between model lightweighting and real-time performance remains a challenge, especially when deployed on resource-constrained maritime monitoring equipment.
3. The fine-grained detection capabilities for the type, size, and posture of maritime small targets still need to be improved, which is crucial for the accurate recognition and classification of maritime small targets [20].

To address these problems, we propose a maritime small target image detection algorithm based on the improved YOLOv11n, which includes three innovations:

1. Bilateral Information Interaction (BIE) Module: The BIE module adopts a dual-branch information interaction design, focusing on the thermal radiation semantic features of infrared images and the texture detail features of visible light images. Afterwards, the dual-channel information fully interacts to dynamically suppress background interference (such as clouds, fog, and sea surface reflection) and amplify the key feature information of small maritime targets.
2. Novel Lightweight Convolution (C3k2-RepViTBlock): RepViTBlock is combined with the C3k2 module of YOLOv11n to form C3k2-RepViTBlock. By leveraging the structural reparameterization technology of RepViTBlock, the feature processing branch of C3k2 is enhanced, and the key features of small targets are strengthened through local self-attention, ensuring the lightweight nature of the model while achieving the real-time detection of maritime small targets.
3. Convolutional Self-Attention (ConvAttn) Module: The ConvAttn module is introduced into the backbone network. With the adaptive perception of input features by its dynamic small-kernel convolution, it strengthens the weak texture details of ship targets in the tail features of the backbone network and improves the model’s ability to extract features of small ship targets.

## 2. Methods

### 2.1. Model Structure Based on Improved YOLOv11n

**Figure 1:**
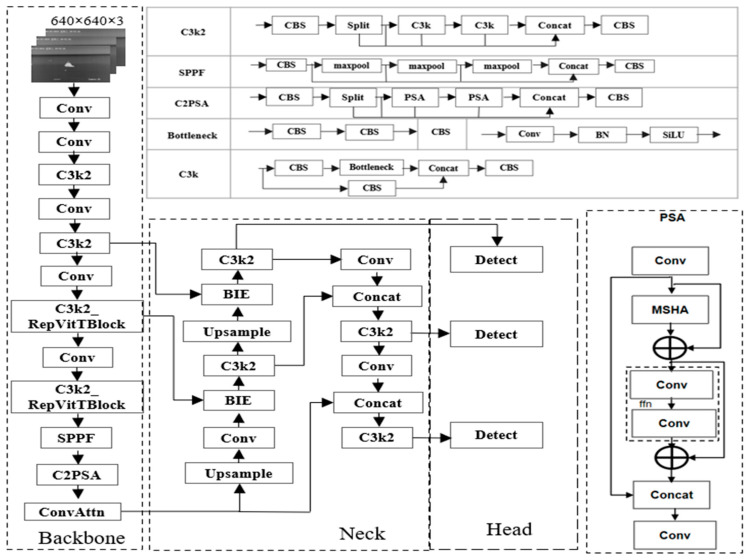
The improved YOLOv11n network structure.

To enhance the detection accuracy and efficiency for small ship targets in infrared and visible light datasets, this study conducts targeted improvements based on YOLOv11n [21]. The structure diagrams of the improved YOLOv11n and its respective modules are shown in Figure 1. YOLOv11n is mainly composed of three parts—the Backbone (backbone network), Neck (neck network), and Head (detection head)—where “conv” denotes the convolution operation. The C3k2 module is improved from C3k. Specifically, CBS consists of Conv (convolution layer), BN (batch normalization), and SiLU (activation function); Bottleneck is composed of two CBS components; C3k is formed by Bottleneck, CBS, and Concat; and the C3k2 module is composed of CBS, C3k, and Concat. To optimize the small target feature extraction capabilities of the C3k2 module, the C3k2-RepViTBlock module replaces C3k in the C3k2 module with RepViTBlock. This modification not only maintains the lightweight property of C3k2 but also improves the model’s ability to detect small ship targets. SPPF refers to spatial pyramid pooling fast. The C2PSA module is derived by replacing C3k in C3k2 with partial self-attention (PSA). Specifically, PSA first uniformly divides the input features into two parts via 1 × 1 convolution; it then feeds one part of the features into the NPSA block (composed of multi-head self-attention (MHSA) and a feed-forward network (FFN)). Finally, the two groups of features are connected and fused through 1 × 1 convolution. ConvAttn is the convolutional self-attention module. BIE is the bilateral information interaction module, which leverages the advantages of dual-channel feature information interaction to improve the model’s ability to extract features of small ship targets in infrared and visible light datasets. The Neck realizes the integration of high-level semantic features and low-level detailed features, while the Head fulfills functions such as target classification and the prediction of the bounding box size.

### 2.2. Bilateral Information Exchange (BIE) Module

**Figure 2:**
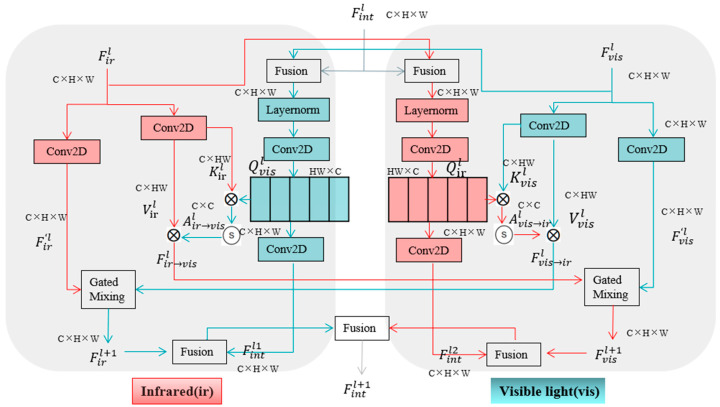
The BIE module network structure.

For the detection of small ship targets in infrared and visible light image datasets, we introduce and improve the BIE module [22] in the Neck network of YOLOv11n. The BIE module is shown in Figure 2. Here, $F_{int}^{l}$, $F_{ir}^{l}$, and $F_{vis}^{l}$ are the upper-layer global input feature, infrared branch feature, and visible light branch feature, respectively. First, $F_{int}^{l}$ is divided into two parts for feature fusion with $F_{ir}^{l}$ and $F_{vis}^{l}$. Then, they are updated through layer normalization and a Conv2D, respectively. After this, queries $Q_{ir}^{l}$ and $Q_{vis}^{l}$ are obtained through a 1 × 1 convolution layer, and then $F_{int}^{l1}$ and $F_{int}^{l2}$ are obtained through a Conv2D. Taking the infrared branch feature in the left part of Figure 2 as an example, the key $K_{ir}^{l}$ and value $V_{ir}^{l}$ are obtained by $F_{ir}^{l}$ through a Conv2D. Subsequently, $K_{ir}^{l}$ and $Q_{ir}^{l}$ are multiplied by matrix operation, and then an attention score matrix $A_{ir\to vis}^{l}$ is obtained through a Softmax function operation, which reflects the semantic correlation between $Q_{ir}^{l}$ and $K_{ir}^{l}$. Then, $A_{ir\to vis}^{l}$ is multiplied by $V_{ir}^{l}$ to obtain $F_{ir\to vis}^{l}$ containing visible light branch features. Similarly, the visible light branch feature in the right part of Figure 2 is used to obtain $F_{vis\to ir}^{l}$ containing infrared features. After this, $F_{ir\to vis}^{l}$ and $F_{vis}^{l}$ obtained through Conv2D are dynamically fused through a gating mechanism to output the updated $F_{vis}^{l+1}$. Similarly, $F_{ir}^{l+1}$ is obtained. $F_{vis}^{l+1}$ and $F_{int}^{l2}$ are feature-fused, $F_{ir}^{l+1}$ and $F_{int}^{l1}$ are feature-fused, and finally the two obtained groups of features are fused to output new feature information $F_{int}^{l+1}$. The formulas of the BIE module are shown in Equations (1)–(7).

(1) $$F_{ir\to vis}^{l}={Conv}_{1\times 1}(Softmax(Q_{vis}^{l}{\left(K_{ir}^{l}\right)}^{T}/\sqrt{HW})V_{ir}^{l})$$

(2) $$F_{vis\to ir}^{l}={Conv}_{1\times 1}(Softmax(Q_{ir}^{l}{\left(K_{vis}^{l}\right)}^{T}/\sqrt{HW})V_{vis}^{l})$$

Among them, $F_{ir\to vis}^{l}$ is the feature containing visible light information, and $F_{vis\to ir}^{l}$ is the feature containing infrared information. $Q_{ir}^{l}$, $K_{ir}^{l}$, $V_{ir}^{l}$, and $Q_{vis}^{l}$, $K_{vis}^{l}$, $V_{vis}^{l}$ are, respectively, the queries, keys, and values of infrared and visible light image features. H and W are the height and width.

(3) $$F_{ir}^{l+1}=Z_{ir}^{l}⊙F_{ir}^{l}+(1-Z_{ir}^{l})⊙F_{vis\to ir}^{l}$$

(4) $$Z_{ir}^{l}=\sigma (W_{n1}F_{ir}^{l}+W_{n2}F_{vis\to ir}^{l})$$

(5) $$F_{vis}^{l+1}=Z_{vis}^{l}⊙F_{vis}^{l}+(1-Z_{vis}^{l})⊙F_{ir\to vis}^{l}$$

(6) $$Z_{vis}^{l}=\sigma (W_{n1}F_{vis}^{l}+W_{n2}F_{ir\to vis}^{l})$$

(7) $$F_{int}^{l+1}=F_{fused}(F_{fused}\left(F_{ir}^{l+1},F_{int}^{l1}\right),F_{fused}\left(F_{vis}^{l+1},F_{int}^{l2}\right))$$

Among them, $F_{ir}^{l+1}$ and $F_{vis}^{l+1}$ are the updated features, $Z_{ir}^{l}$ and $Z_{vis}^{l}$ are gating coefficients, $W_{n1}$ and $W_{n2}$ are weight coefficients, ⊙ is element-wise multiplication, σ is the Sigmoid function, $F_{fused}(.)$ is weighted feature fusion, and $F_{int}^{l+1}$ is the final output feature.

The BIE module solves the problem of single-modality feature information loss in infrared and visible light image datasets. Meanwhile, it addresses the difficulty in extracting the global structures of small ships and long-distance vessels. It fully integrates global contextual information features. Its internal gating mechanism dynamic fusion module not only retains the original ship features but also supplements the inter-modality global structure, outputting more robust fused features. For possible sea clutter and environmental interferences in ship images (such as the impact of clouds and fog on infrared images and the impact of water surface reflection on visible light images), the BIE module is used to screen effective features and suppress misleading interferences, significantly improving the detection capabilities of YOLOv11n for small-target ships.

### 2.3. A Three-Scale Convolution Dual-Path Variable-Kernel Module Based on RepViTBlock (C3k2-RepViTBlock)

**Figure 3:**
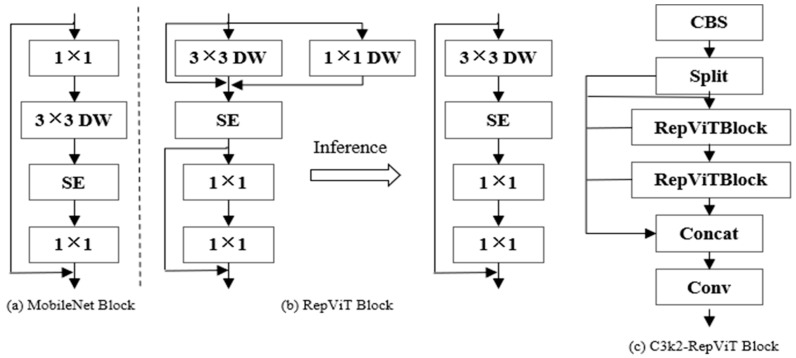
The C3k2-RepViTBlock module network structure.

To improve the ability of the C3k2 module in YOLOv11n to capture fine-grained spatial information and long-range dependencies, while a pure Transformer module has a large computational overhead and is not conducive to the feature perception of small target ships, we combine the C3k2 module of YOLOv11n with the RepViTBlock module [23] to form C3k2-RepViTBlock. C3k2-RepViTBlock is composed by replacing the C3 module in C3k2 with RepViTBlock. While retaining the multi-branch residual structure of the C3 module, it enhances the model’s ability to capture the spatial details and global semantic associations of small targets. The structure diagram of the C3k2-RepViTBlock module is shown in Figure 3c.

The RepViTBlock module adopts a dual-branch structure consisting of a token mixer and a channel mixer. The RepViTBlock module is simulated by a MobileNet block. The MobileNet block uses 1 × 1 expansion convolution and a 1 × 1 projection layer to realize inter-channel interaction (i.e., channel mixer). Then, a 3 × 3 depthwise separable convolution (DW) is added to perform spatial convolution independently for each channel. The channel dimension is weight-calibrated through the squeeze-and-excitation (SE) layer and 1 × 1 convolution (i.e., token mixer), enhancing the model’s key channel features for small targets. The MobileNet block is shown in Figure 3a. To separate the token mixer and channel mixer, RepViTBlock moves the DW convolution and SE layer upward. It adopts multi-scale convolution branches in the training phase and merges the multi-branches into a single branch through reparameterization technology in the inference phase. This significantly improves the detection accuracy for small vessels and the inference efficiency of edge devices. The structure diagram of the RepViTBlock module is shown in Figure 3b.

Considering the discrepancies in feature representation across different hierarchical levels of the YOLOv11n backbone network, the C3 modules within the first two shallow C3K2 blocks of the YOLOv11n backbone network are retained. This hierarchical level primarily extracts detailed features (e.g., textures and edges) of maritime targets, where excessive enhancement would amplify background interferences such as ocean waves and cloud/fog noise. In contrast, the C3 modules in the last two deep C3K2 blocks of the YOLOv11n backbone network are modified by substituting them with RepViTBlock modules. As a critical component in fusing detailed features and global semantic features, this deep hierarchical level leverages the dual-branch structure (consisting of the token mixer and channel mixer) of the RepViTBlock module, combined with the residual structure of C3K2, to synergistically enhance the model’s capabilities in extracting the global semantic features of small maritime targets.

### 2.4. Convolutional Self-Attention Module (ConvAttn)

**Figure 4:**
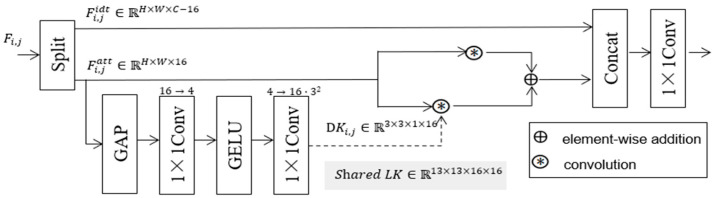
The ConvAttn module network structure.

Due to the large computational overhead of traditional attention modules and the insufficient modeling abilities of convolution modules for long-range feature dependencies, we introduce the ConvAttn module [24] at the end of the backbone network of YOLOv11n. It adopts shared long-range convolution kernels to enhance the model’s detection abilities for long-distance small target ships. The structure diagram of the ConvAttn module is shown in Figure 4. First, the input feature map $F_{i,j}$ is split along the channel dimension to obtain the attention branch feature $F_{i,j}^{att}$ and the identity mapping branch feature $F_{i,j}^{idt}$, which are, respectively, used for subsequent attention weight calculation and multi-scale fusion. For the attention branch $F_{i,j}^{att}$, global information compression is performed on $F_{i,j}^{att}$ through global average pooling (GAP) to capture global features in the channel dimension. Then, two layers of 1 × 1 convolution are used to generate kernel parameters adapted to 3 × 3 depthwise convolution. After this, a dynamic depthwise convolution kernel (${DK}_{i,j}$) is generated through a nonlinear activation function (GELU) to enhance the feature expression ability. Then, $F_{i,j}^{att}$ is element-wise multiplied by ${DK}_{i,j}$ and ${LK}_{i,j}$ (shared long-range convolution kernels), respectively, and the two results are added to obtain $F_{i,j}^{rena}$. Finally, $F_{i,j}^{rena}$ and $F_{i,j}^{idt}$ are concatenated, and then a 1 × 1 convolution is applied to realize multi-scale feature fusion to obtain the final output $F_{i,j}^{fuse}$. The formulas of the ConvAttn module are shown in Equations (8)–(11).

(8) $$F_{i,j}^{att},F_{i,j}^{idt}={Split}_{16:C-16}(F_{i,j})$$

(9) $${DK}_{i,j}={Conv}_{1\times 1}^{up}(\phi ({Conv}_{1\times 1}^{down}(GAP(F_{i,j}^{att}))))$$

(10) $$F_{i,j}^{res}=\left(F_{i,j}^{att}⊛{DK}_{i,j}\right)+\left(F_{i,j}^{idt}⊛{LK}_{i,j}\right)$$

(11) $$F_{i,j}^{fuse}={Conv}_{1\times 1}^{fuse}(Concat(F_{i,j}^{res},F_{i,j}^{idt}))$$

Among them, (i, j) are the position coordinates of elements in the feature map, $F_{i,j}^{att}$ is the attention branch feature, $F_{i,j}^{idt}$ is the identity mapping branch feature, ${DK}_{i,j}$ is the dynamic depthwise convolution kernel, ${Conv}_{1\times 1}^{up}(.)$ is the upsampling convolution, ${Conv}_{1\times 1}^{down}(.)$ is the downsampling convolution, ϕ is the activation function, ⊛ is the convolution operation, Concat is the channel concatenation operation, and $F_{i,j}^{fuse}$ is the multi-scale feature fusion feature.

The ConvAttn module utilizes the dynamic depthwise convolution kernel (${DK}_{i,j}$) to extract the fine-grained features of small targets (such as tiny components of small ships) and ${LK}_{i,j}$ (shared long-range convolution kernel) to fuse cross-modal semantic information (the global association between infrared thermal contours and visible light shapes). This solves the problems whereby small targets are easily obscured by the background and cross-modal feature fusion is insufficient. In addition, compared with the self-attention module, the ConvAttn module significantly reduces the number of model parameters through ${LK}_{i,j}$ (shared long-range convolution kernel) and improves the operating efficiency of the model.

## 3. Experimental Results and Analysis

### 3.1. Experimental Environment and Parameter Configuration

The experimental environment has a crucial impact on the efficiency and accuracy of model training. A scientific and reasonable configuration can realize the optimal utilization of hardware resources, improve the training efficiency, and ensure the repeatability and reliability of the experimental results. We summarize the specific software and hardware configurations adopted in the experiment in Table 1.

The configuration of the hyperparameters is also of key significance to the performance and generalization ability of the model. Scientific tuning can improve the learning efficiency, increase the detection accuracy, and reduce the degree of overfitting, thereby enabling the model to effectively adapt to detection in long-distance small target scenarios. The details of the hyperparameters adopted in our experiment are shown in Table 2 below.

### 3.2. Dataset

This study uses two types of datasets. The first is a dataset (IVships) collected by visible light and infrared acquisition devices, which contains two types of images—infrared and visible light—covering 7 categories of targets; specifically, these are cargo ships, ferries, coast guard ships, buoys, fishing boats, tugboats, and rescue ships. The dataset contains a total of 6518 images, with 4563 images in the training set, 1304 images in the validation set, and 651 images in the test set. The dataset information is shown in Table 3.

The second is a public dataset (SeaShips), which is mainly composed of visible light images and includes 6 categories: bulk cargo carriers, container ships, fishing boats, general cargo ships, ore carriers, and passenger ships. Details of this dataset are shown in Table 4.

### 3.3. Experimental Evaluation Metrics

This experiment adopts the mean average precision (mAP, with an IoU threshold of 0.5) as the evaluation metric for algorithm performance. The formulas for precision (P) and recall (R) are as follows:

(12) $$P=\frac{TP}{\left(TP+FP\right)}$$

(13) $$R=\frac{TP}{TP+FN}$$

where TP is the number of ships correctly predicted as positive examples by the model, FP is the number of ships incorrectly predicted as positive examples by the model, and FN is the number of ships incorrectly predicted as negative examples by the model.

Average precision (AP) is the area under the P-R curve. The mAP is obtained by averaging the AP values calculated for all categories. The formulas for AP and mAP are as follows:

(14) $$\begin{array}{c} AP=\int_{0}^{1}PRdR \end{array}$$

(15) $$mAP=\frac{1}{n}\sum_{i=1}^{n}AP_{i}$$

where P(R) is the *p* value when the abscissa is R in the P-R curve, and n is the number of target categories in the dataset.

### 3.4. Ablation Experiment

As shown in Table 5, to verify the effectiveness of the improvement, we conducted ablation experiments based on YOLOv11n on the collected IVships dataset and the public SeaShips dataset, so as to evaluate the enhancement effects of each module on the model. “×” denotes non-inclusion, and “√” denotes inclusion.

It can be seen from the comparison results in Table 5 that, in the two datasets of IVships and SeaShips, after YOLOv11n introduces BIE, the mAP@0.5 values increase by 1.4% and 1.1%, respectively. This module efficiently extracts features and realizes information complementation through the bilateral information interaction mechanism. After YOLOv11n adds C3k2-RepViTBlock, the mAP@0.5 values increase by 0.6% and 0.2%, respectively, and its dual-branch structure composed of a token mixer and channel mixer greatly improves the cross-modal ship detection accuracy. After YOLOv11n introduces ConvAttn in the backbone network, the mAP@0.5 values reach 86.8% and 98.3%, which are 0.3% and 1% higher than those of the original YOLOv11n, respectively; this feature enhancement module realizes multi-scale feature fusion by using shared long-distance convolution kernels. In addition, when C3k2-RepViTBlock and BIE are introduced simultaneously, the mAP@0.5 values are 1.6% and 1.2% higher than those of YOLOv11n, respectively. Compared with the original model, YOLOv11n, the improved algorithm increases the mAP@0.5 values by 1.9% and 1.7%, respectively, and the precision by 2.2% and 2.4%, respectively, significantly enhancing the model’s small target detection capabilities.

### 3.5. Comparative Experiment

#### 3.5.1. Comparative Experiment with Mainstream Algorithms

To further demonstrate the advancement of the improved YOLOv11n model, we conducted a comparative experiment by comparing the algorithm presented in this paper with other mainstream object detection algorithms (Faster-RCNN, YOLOv10n, YOLOv8n, YOLOv5s, etc.). The comparative experiment is shown in Table 6. It can be seen from Table 6 that the algorithm introduced in this paper outperforms other advanced algorithms in terms of the mAP@0.5. Since the overall number of parameters of the improved algorithm is increased compared with the original algorithm, the training time and FLOPs are higher than those of the original algorithm but lower than those of most advanced algorithms, and the FPS is better than that of the original algorithm. This ensures the real-time performance and light weight of the model.

#### 3.5.2. Performance Analysis of Object Detection for Each Category

We now conduct a refined performance analysis for each object category. As shown in Table 7, we compare the AP (%) of the original model and the improved model for each single object category to evaluate the improvement effect and detection consistency in specific categories.

As can be seen from Table 7, the AP values of the improved YOLOv11n have been stably improved for all maritime object categories. Especially in the IVships dataset, the improvement effects on buoys, tugboats, and rescue ships are relatively significant. Compared with the original model, they are increased by 3.6%, 2.4%, and 2.4%, respectively. This indicates that the detection performance of the model for small maritime targets has been significantly enhanced.

## 4. Visualization Analysis

### 4.1. Analysis of Test Images

**Figure 5:**
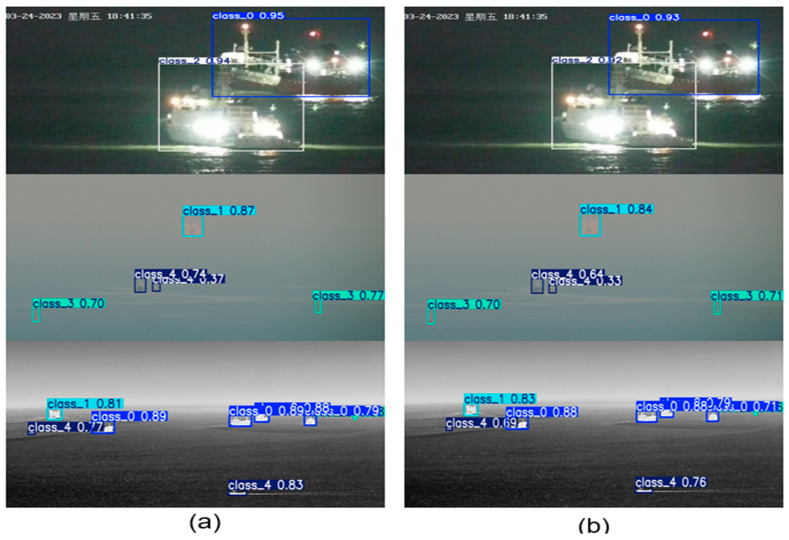
Comparative test images of the improved algorithm and the original algorithm: (**a**) test image of the improved YOLOv11n and (**b**) test image of YOLOv11n.

To verify the performance of the improved algorithm under infrared and visible light images, this paper compares the detection performance of the improved algorithm and the original algorithm under different images. We take the images in the IVships dataset as an example. The test results of the improved YOLOv11n are shown in Figure 5a, and the test results of the original YOLOv11n algorithm are shown in Figure 5b. The visible light images in the first row were captured in a low-light scene at night. The ship lights are bright and there is obvious light and shadow interference on the sea surface, which easily affects the normal detection and classification of the model. The visible light images in the second row were captured in a scene obscured by clouds and fog. Affected by cloudy and foggy weather, small maritime targets have poor overall light penetration and loss of visual information, which makes the model detection difficult. The infrared images in the third row were captured in a night-time open-sea scene, with multiple types of targets distributed in a scattered manner, some targets occluded, and a large number of small targets, making model detection extremely difficult. As shown in Figure 5, the improved model has higher detection confidence and more accurate bounding box selection for targets in various complex scenarios. At the same time, it solves the problems of missed detection and the insufficient confidence of the original model in small targets and low-light/foggy scenarios, and the small target detection capabilities are significantly improved.

### 4.2. Heatmap Analysis

**Figure 6:**
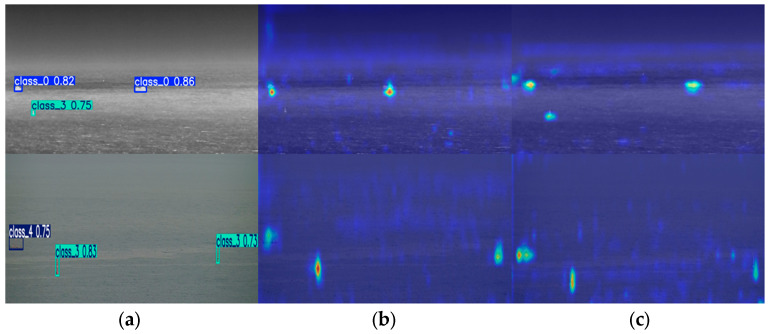
Original image and heatmap: (**a**) original image, (**b**) pre-improvement algorithm, and (**c**) post-improvement algorithm.

Heatmaps can indicate information such as target positions and confidence levels through color intensity, providing an intuitive visualization of the model’s attention to different targets and its detection results. We take the images in the IVships dataset as an example. The original images and the heatmaps of the model (before and after improvement) are shown in Figure 6.

As can be seen from Figure 6b,c, under both infrared and visible light images, the improved model’s heatmaps can almost fully cover the targets—whether they are long-distance cargo ships or small targets like buoys and fishing boats in cloudy and foggy environments. Meanwhile, it reduces the interference of irrelevant backgrounds on the model. In contrast, the original model exhibits missed detections: it fails to detect the buoy (class_3) in the infrared image, and its heatmap does not fully cover the fishing boat (class_4) in the visible light image. This further demonstrates the feasibility of the improved model proposed in this paper.

## 5. Conclusions

This paper proposes a small maritime target detection algorithm based on the improved YOLOv11n. Specifically, the BIE module is introduced into the neck network of the algorithm, and its bilateral information interaction mechanism is utilized to realize the fusion of small target features under infrared and visible light images, thereby enhancing the feature extraction capabilities of the model. The C3k2 module in the backbone network is improved to C3k2-RepViTBlock; on the premise of maintaining the light weight of the model, reparameterization technology is adopted to improve the detection accuracy of the model for small maritime targets. Finally, the ConvAttn module is added to the backbone network, which uses dynamic depth convolution and long-distance convolution to enhance the model’s ability to detect small targets at long distances. Extensive experiments conducted on infrared and visible light image datasets verify the effectiveness of the improved algorithm proposed in this paper. Compared with the original algorithm, the improved algorithm increases the mAP@0.5 values by 1.9% and 1.7% in the IVships and SeaShips datasets, respectively, and increases the average precision by 2.2% and 2.4%, respectively. Although the improved YOLOv11n has achieved a significant improvement in detection performance for small maritime targets under complex scenarios (such as night-time low-illumination, cloud and fog occlusion, and open-sea infrared scenes), the algorithm still has the problems of an increased parameter quantity and computational cost. In the future, we will continue to explore structural pruning and quantization strategies for the improved model to reduce the parameter quantity and computational cost while maintaining the detection accuracy. On the other hand, we will expand the dataset to cover more extreme maritime scenarios to further optimize the generalization ability and robustness of the algorithm.

## Figures and Tables

**Table 1:** Environment setup for experiments.

Environment Component	Configuration Details
Operating System	Windows 11
GPU	NVIDIA GeForce RTX 4060 (NVIDIA, Santa Clara, CA, USA)
CPU	13th Gen Intel^®^ CoreTM i7-13650HX (Intel Corporation, Santa Clara, CA, USA)
CUDA Version	Cuda 12.6
Framework	PyTorch2.0.1
Programming Language	Python 3.9

**Table 2:** Experimental parameter settings.

Parameter Name	Value
Batch Size	16
Number of Epochs	300
Input Image Size	640 × 640
Optimizer	Adam
Initial Learning Rate	0.01
Momentum	0.937
Weight Decay	0.0005

**Table 3:** IVships dataset.

Category	Quantity
Cargo ship (class_0)	9382
Ferry (class_1)	2554
Coast guard ship (class_2)	613
Buoy (class_3)	4017
Fishing boat (class_4)	7356
Tugboat (class_5)	491
Rescue ship (class_6)	489

**Table 4:** SeaShips dataset.

Category	Quantity
Bulk cargo carrier (class_0)	1952
Container ship (class_1)	901
Fishing boat (class_2)	2190
General cargo ship (class_3)	1505
Ore carrier (class_4)	2199
Passenger ship (class_5)	474

**Table 5:** Results of ablation experiments.

Dataset	Baseline Algorithm	BIE	C3k2-RepViTBlock	ConvAttn	P (Precision)/%	R (Recall)/%	mAP@0.5/%
IVships	YOLOv11n	×	×	×	88.7	80.3	86.5
		√	×	×	89.2	82.5	87.9
		×	√	×	88.8	81.5	87.1
		×	×	√	88.4	81.4	86.8
		√	√	×	89.8	82.4	88.1
		√	√	√	90.9	82.9	88.4
SeaShips		×	×	×	95.7	96.5	97.2
		√	×	×	96.3	97.4	98.4
		×	√	×	95.8	96.5	97.5
		×	×	√	96.8	96.9	98.3
		√	√	×	97.6	96.5	98.5
		√	√	√	98.1	97.1	98.9

**Table 6:** Comparison experiment.

Baseline Algorithm	Parameters (M)	FLOPs/10^9^	FPS (fr/s)	Dataset	mAP@0.5/%	Training Duration (h)
YOLOv11n	2.58	6.3	24.7	IVships/SeaShips	86.5/97.2	12.5/16.5
YOLOv10n	2.71	6.5	22.8		84.5/96.1	18.8/19.2
YOLOv8n	3.01	8.1	26.3		83.5/96.5	17.3/20.3
YOLOv7tiny	6.01	12.8	21.5		84.1/96.4	20.5/21.5
YOLOv5s	6.82	14.5	20.5		81.2/95.3	21.3/23.2
Faster-RCNN	25.63	65.1	8.2		71.2/88.1	25.5/27.2
Improved YOLOv11n	3.63	14.2	26.8		88.4/98.9	13.3/18.6

**Table 7:** Analysis of detection results for different maritime target categories.

Dataset	Ship Category	Algorithm	AP/%
IVships/SeaShips	Cargo ship/Bulk cargo carrier (class_0)	YOLOv11n	97.4/96.5
		Improved YOLOv11n	98.5/99.2
	Ferry/container ship (class_1)	YOLOv11n	97.8/96.5
		Improved YOLOv11n	98.5/99.5
	Coast guard ship/fishing boat (class_2)	YOLOv11n	98.2/96.8
		Improved YOLOv11n	99.5/97.9
	Buoy/general cargo ship (class_3)	YOLOv11n	75.3/98.8
		Improved YOLOv11n	78.9/99.5
	Fishing boat/ore carrier (class_4)	YOLOv11n	51.5/97.4
		Improved YOLOv11n	53.3/98.8
	Tugboat/passenger ship (class_5)	YOLOv11n	92.3/97.3
		Improved YOLOv11n	94.7/98.8
	Rescue ship (class_6)/	YOLOv11n	93.0/
		Improved YOLOv11n	95.4/

## Data Availability

Data are contained within the article.
